# Graphene quantum dot-crafted nanocomposites: shaping the future landscape of biomedical advances

**DOI:** 10.1186/s11671-024-04028-2

**Published:** 2024-05-02

**Authors:** Mohammad Suhaan Dar, Niroj Kumar Sahu

**Affiliations:** Centre for Nanotechnology Research, Vellore Institute of Technology, Vellore, Tamil Nadu, 632014 India

**Keywords:** Nanocomposites, GQDs, Cancer, Theranostics, Nanomedicine

## Abstract

Graphene quantum dots (GQDs) are a newly developed class of material, known as zero-dimensional nanomaterials, with characteristics derived from both carbon dots (CDs) and graphene. GQDs exhibit several ideal properties, including the potential to absorb incident energy, high water solubility, tunable photoluminescence, good stability, high drug-loading capacity, and notable biocompatibility, which make them powerful tools for various applications in the field of biomedicine. Additionally, GQDs can be incorporated with additional materials to develop nanocomposites with exceptional qualities and enriched functionalities. Inspired by the intriguing scientific discoveries and substantial contributions of GQDs to the field of biomedicine, we present a broad overview of recent advancements in GQDs-based nanocomposites for biomedical applications. The review first outlines the latest synthesis and classification of GQDs nanocomposite and enables their use in advanced composite materials for biomedicine. Furthermore, the systematic study of the biomedical applications for GQDs-based nanocomposites of drug delivery, biosensing, photothermal, photodynamic and combination therapies are emphasized. Finally, possibilities, challenges, and paths are highlighted to encourage additional research, which will lead to new therapeutics and global healthcare improvements.

## Introduction

Worldwide, cancer and cardiovascular diseases are increasingly becoming the leading factors contributing to mortality. Reports indicate that in 2020, there were approximately 10 million global fatalities and 18.1 million reported cases of cancer. Simultaneously, cardiovascular diseases are accountable for an estimated 32% of total global deaths [1]. Recognizing the severity of these pathophysiologies and the significant increase in their prevalence, it is imperative to undertake extensive research endeavours to develop advanced biomedical tools for addressing these global health challenges.

**Fig. 1:**
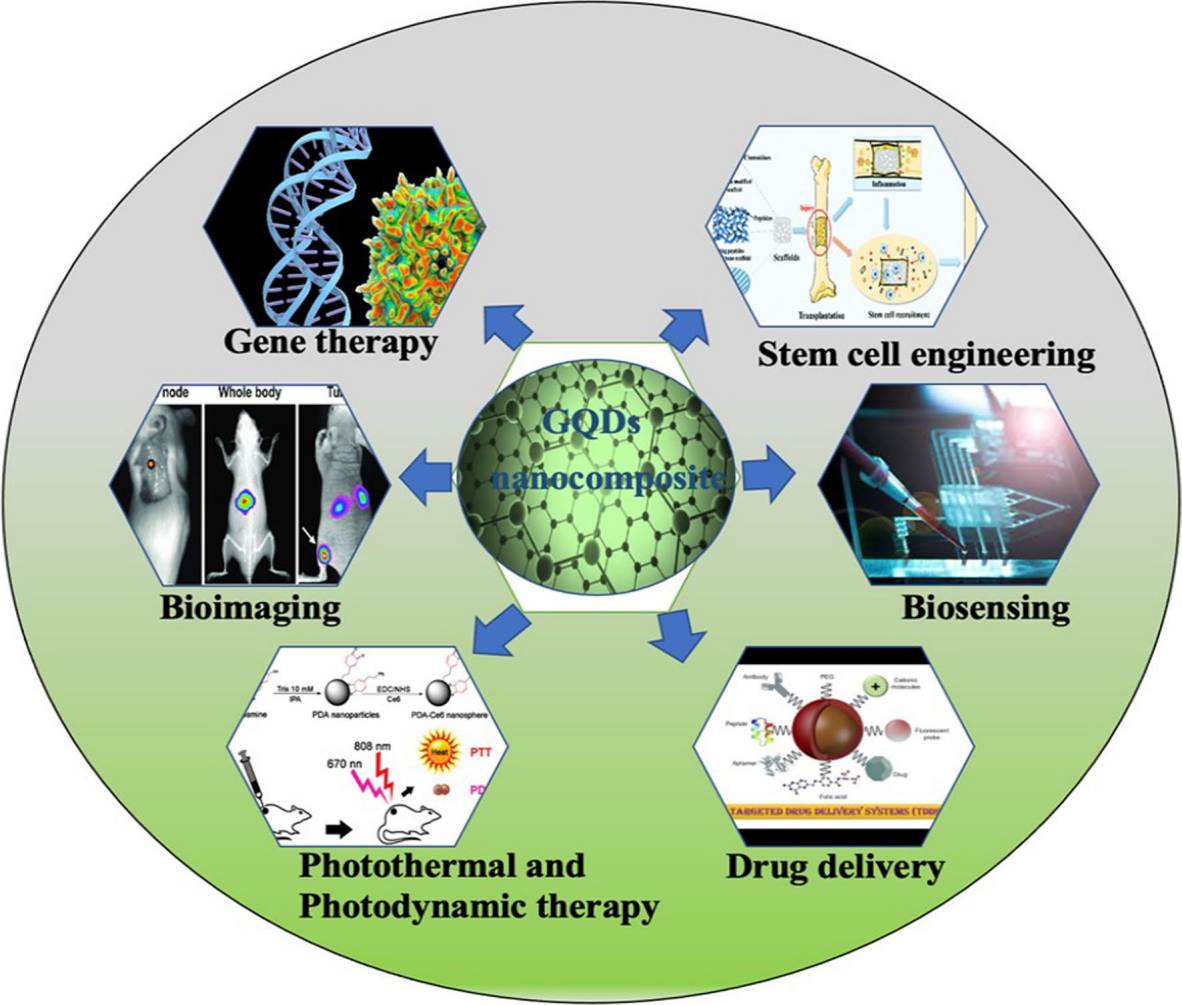
Biomedical applications of multifunctional GQDs-based nanocomposites

GQDs have made significant strides in the field of nanomaterials, witnessing continuous expansion in research. Notably, GQDs-based nanocomposites have garnered increased attention from scientists in recent years [2]. GQDs have gained popularity due to their exceptional biological characteristics, including biocompatibility, chemical stability, high surface area-to-volume ratios, and transparency. Moreover, their dispersibility in various solvents and favorable solubility characteristics, both in aqueous and organic solvents, make them highly versatile for integration into diverse systems for targeted applications. Additionally, GQDs exhibit electrical conductivity and inertness, further enhancing their utility across a wide range of fields [3]. The application of GQDs-based nanocomposites has emerged as a ground-breaking technology in various scientific domains such as photonics, composite materials, energies, and optoelectronics, experiencing rapid growth. GQDs-based nanomaterials have demonstrated remarkable potential in the biomedical field, playing pivotal roles in nano-theranostics, diagnostics, drug delivery, near-infrared light (NIR)-induced photothermal and photodynamic treatments, bioimaging, gene therapy, biosensors, and more. The versatility and promising applications of GQDs-based nanocomposites make them a focal point for advancing scientific frontiers, especially in the realm of biomedical research [4–6]. Figure 1 depicts the various biomedical applications of nanocomposites based on multidimensional GQDs.

The central objective of this review is to provide the scientific community with a foundational understanding of GQDs-based nanocomposites, encompassing their classification, synthesis, applications, and utilization within the biomedical domain. The primary aim is to elucidate their potential in addressing a spectrum of severe diseases. By exploring the intricacies of GQDs-based nanocomposites, we seek to contribute to the knowledge base, offering insights that can pave the way for innovative strategies in combating some of the most formidable health challenges.

## Historical perspective of GQDs in biomedical sector

Exploratory research, driven by curiosity, has yielded transformative discoveries, exemplified by innovations like buckyballs and graphene [7]. In the contemporary landscape, carbon-based materials are playing an increasingly pivotal role with profound implications. Renowned British scientist Harry Kroto and collaborators were awarded the 1996 Nobel Prize in Chemistry for their groundbreaking work on carbon "buckyballs," consisting of just 60 carbon atoms. This breakthrough paved the way for the development of remarkably small and swift computers [8, 9]. In a subsequent milestone, the 2010 Nobel Prize in Physics was awarded to Andre Geim and Konstantin Novoselov for their pioneering discovery of graphene, the foundational structure for all graphitic forms. Graphene has emerged as an exceptionally promising nanomaterial due to its unparalleled combination of properties [10]. Furthermore, recent progress in quantum dot research has significantly elevated their importance. Notably, the recognition of achievements in the field, akin to Nobel Prizes, underscores the transformative impact and potential of quantum dots, adding a new dimension to the evolving landscape of nanomaterials.

**Fig. 2:**
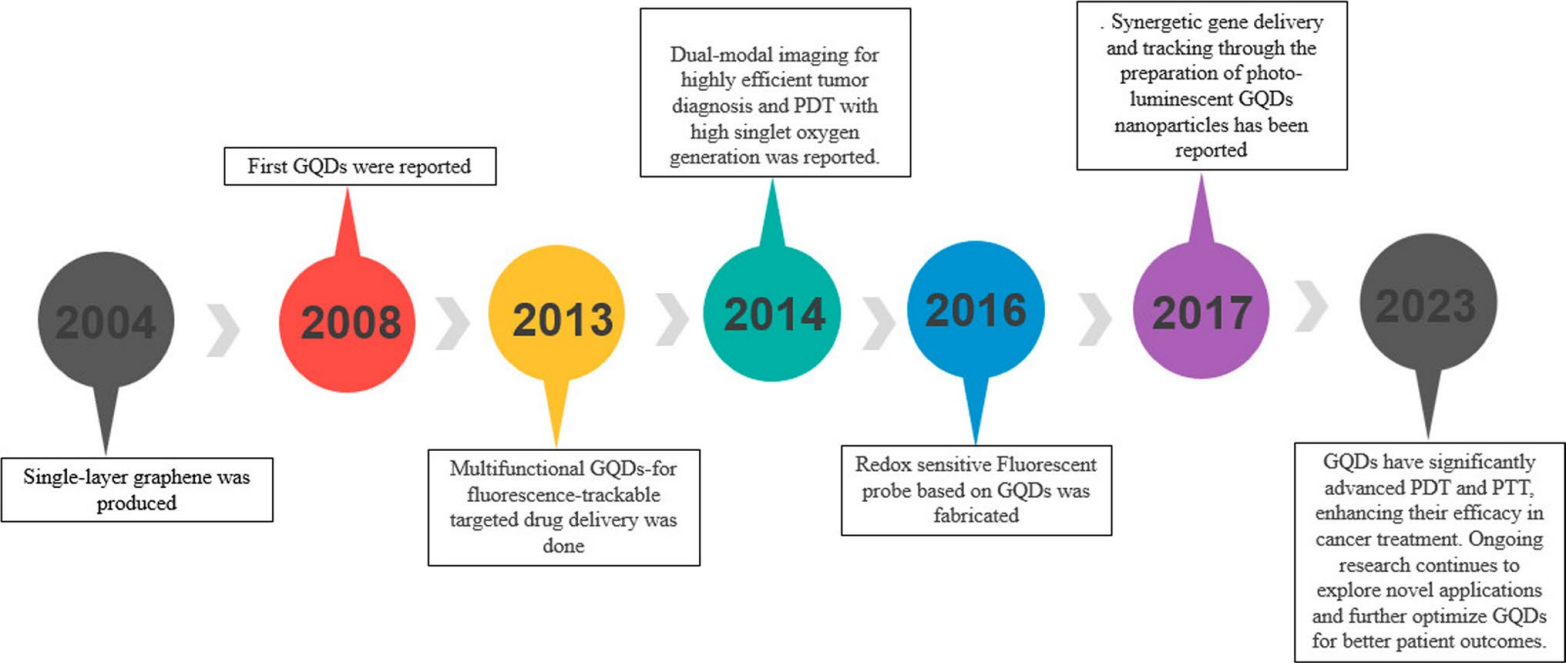
Journey of GQDs in biomedical applications

**Fig. 3:**
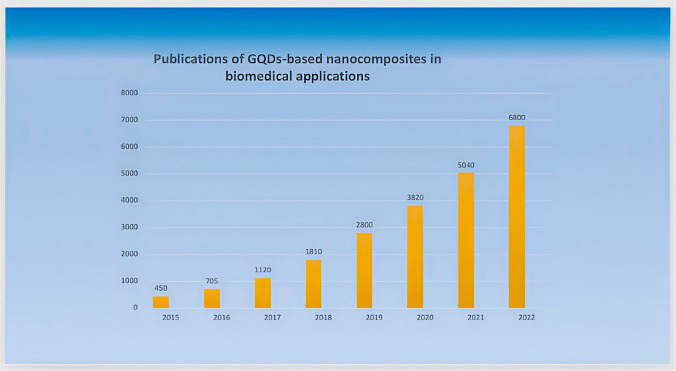
Number of scientific publications per year related to GQDs-based nanocomposites for biomedical applications. Data obtained from Web of Science accessed in September 2023 using the words “GQDs nanocomposites in biomedical applications”

The characteristics and applications of graphene have undergone extensive exploration since its discovery. However, researchers have identified several limitations associated with graphene, such as zero bandgaps, hydrophobicity, and challenges in large-scale production [11, 12]. To overcome these drawbacks, researchers have delved into the realm of structural modification of graphene. Ponomarenko et al. [13] expanded on previous investigations into CDs and introduced GQDs depicted in Fig. 2. Unlike CDs, GQDs feature dots within the graphene lattice that are thinner than 10 layers and smaller than 100 nm, setting them apart. GQDs, a sole member of the carbon family with a zero-dimensional structure, are composed of one to several layers of graphene sheets with lateral dimensions of less than 10 nm [14]. The distinctions between CDs and GQDs will be thoroughly examined in the preceding section. The evolution of GQDs in the biomedical domain is marked by their efficiency in various applications, owing to their exceptional chemical, physical, and biological characteristics. In 2013, Zheng X and colleagues devised a successful approach for fluorescence imaging and targeted drug delivery, showcasing the capability of GQDs to reveal the intracellular location of nanocarriers through fluorescence. This revealed that active endocytosis serves as the mechanism of uptake [15]. Building on this progress, in 2014, Huang C and co-workers introduced a GQDs-based nanocomposite designed for dual-modality bioimaging and tumor-targeted drug delivery. This composite exhibited limited cytotoxicity and exceptional biocompatibility within specified concentration ranges [16]. Another notable advancement occurred in 2016 when Liu H. and team developed a redox-modulated fluorescence method using GQDs for the detection of glucose and uric acid [17]. In 2017, Ghafary and collaborators contributed to the field by creating outstanding GQDs nanocarriers for simultaneous gene delivery and tracking. Their work demonstrated improved nuclear internalization and tracking of plasmid (pDNA) both in vitro and in vivo [18]. Additionally, GQDs-based nanocomposites have recently demonstrated promising results in photothermal (PTT) and photodynamic therapy (PDT) in both in vitro and in vivo settings. However, there is a need for further research to effectively address limitations related to renal clearance, size, quantum yield, and toxicity [19, 20]. Figure 3 illustrates the upward trend in scientific articles on GQDs composites in recent years. The research community is actively engaged in efforts to control size, incorporate dopants, and utilize different polymers to enhance GQDs for diverse applications, underscoring the dynamic nature of ongoing investigations in this field.

## Overview of CDs and GQDs

CDs and GQDs are two examples of advanced carbon-based nanomaterials that have useful biomedical applications. These materials are naturally versatile, low-cost, biocompatible, have a large specific surface area, excellent electrical properties, are chemically stable, have low toxicity, and have abundant functionalization sites along the material's edges. Their adaptability to different surface chemistries and nanomaterial modifiers increases their potential applications [21, 22].

### Differences in structure and synthesis of GQDs and CDs

GQDs and CDs exhibit notable differences in their structural properties and synthesis methods. GQDs are crystalline and Sp^2^ hybridised, while CDs are Sp^2^ hybridized carbon domains with Sp^3^ hybridized amorphous carbons. CDs appear luminescent due to mutual effect or collision between surface imperfections and intrinsic core state emissions, while GQDs fluoresce due to quantum confinement. Additionally, GQDs contain graphene sheets within the dots, distinguishing them from CDs, which are typically quasi-spherical carbon nanoparticles [23, 24]. The methodologies employed in the creation of CDs and GQDs exhibit notable variations. Typically, CDs are subjected to top-down methodologies, which involve the chemical, electrochemical, or physical degradation of carbon black or graphene oxide. In contrast, the production of GQDs involves bottom-up approaches, such as the carbonization or pyrolysis of organic substances, or the step-by-step chemical fusion of aromatic molecules. Consequently, the yield of CDs and GQDs vary from one another. In general, quantity obtained through electrochemical exfoliation and chemical oxidation synthesis tend to be lower, but more control over characteristics than hydrothermal and microwave-assisted synthesis, which have faster reaction rates. Optimising reaction parameters and investigating new pathways can increase CDs yield, while carbonisation and purification can increase GQDs yield. Hydrothermal synthesis favours CDs, although electrochemical exfoliation helps regulate GQDs characteristics despite lower yields. Recent advances have enabled the synthesis of biomass-derived GQDs with a yield of over 84%, the highest yet reported. Barium and nitrogen-doped CDs nanocomposite had high quantum yields (QYs) of 99.6% and 99.3%, respectively, indicating their promise for photoluminescence-intensive applications. To complement the understanding provided in the preceding paragraphs, Table 1 summarize the differences between GQDs, and CDs based on the referenced literature, [25–27] presented below.

**Table 1:** depicts the distinctions between CDs and GQDs

Features	GQDs	CDs
Structure	crystalline and Sp^2^ hybridised	Sp^2^ hybridized carbon domains with Sp^3^ hybridized amorphous carbons
Size	usually less than 50 nm	Typically, a few to tens of nanometers
Photoluminescence	Tunable fluorescence properties	Broad emission spectra
Photostability	Excellent	Good
Synthesis methods	Chemical exfoliation of graphene oxide, hydrothermal, electrochemical	Hydrothermal/solvothermal, microwave-assisted, laser ablation
Advantages	Strong photoluminescence, tunable properties, high surface area	Easy synthesis, low cost, versatile
Disadvantages	Complex synthesis, limited scalability	Less defined structure, variable properties
Synthesis pathway advantages	Allows precise control over size and properties, versatile applications	Simple procedures, easily scalable, low-cost
Synthesis pathway disadvantage	Labor-intensive, requires precise conditions	Variable properties, lack of structural control
Yield	Typically low, depending on synthesis method	Highly variable

## Optical properties of GQDs and detection strategies

The photoluminescence (PL) of GQDs distinguishes them from graphene and ignites interest in their potential applications. GQDs are more resistant to blinking and photobleaching than semiconductor quantum dots. GQDs' small size and bandgap contribute to their PL properties. This structure can be modified by adding dopants and functional groups to the edges. These improvements significantly improve the versatility and utility of GQDs in a variety of areas. The quantum confinement characteristics of zigzag and armchair edges in GQDs influence their PL behavior. GQDs with zigzag edges have a lower bandgap than those with armchair edges, resulting in a blue shift in the emission spectrum. Sp^2^ carbon domain-rich precursors, such as citric acid, are favorable for producing GQDs with desirable PL properties. Quantum confinement effects in GQDs provide a size-dependent band structure, allowing for chemically tunable bio-conjugation via π-π interactions and hydrogen bonding with biological molecules. Because of their strong light absorption, GQDs make excellent light-sensitive sensors in optical detectors. Unsynchronized large-bandgap GQDs offer highly selective UV detection [28–32].

Fluorescence resonance energy transfer (FRET) is a typical biosensing approach. This allows photoexcitation energy transfer from donor to acceptor fluorophores. Increasing interest in GQDs as fluorescent markers in biosensing is due to their high brightness, long fluorescence persistence, and resilience to light fading. Using GQDs and gold nanocomposites as energy donors and acceptors, studies created a simple FRET biosensor to detect the S. aureus gene and found biosensor has low detection threshold and high sequence selectivity. similarly, researchers created nanocomposites using silica SNPs as donors and GQDs as acceptors. FRET efficiency is roughly 78% based on excitation-dependent photoluminescence spectra and decay curves [33, 34]. Unlike FRET nanosurface energy transfer (NSET) transfers energy from a molecule dipole to a metal nanosurface, providing a longer effective distance than FRET. Noble metal nanoparticles and GQDs interact via NSET to quench fluorescence signals, causing an "OFF" state. This phenomenon can help develop cell-based biomolecule sensing methods. A higher signal-to-noise ratio and lower nonspecific adsorption make “OFF–ON” sensors better for diverse applications than “ON–OFF” sensors. GQDs-based nanocomposites have been extensively explored [35]. In a particular investigation, ATP was introduced into GQDs, resulting in an "ON" state that allowed for the tracking of ATP molecules via fluorescent signals [36]. Another study looked at using biofunctionalized GQDs as an energy donor and AuNPs as an energy acceptor to detect cancer biomarkers [37].

## Synthesis and surface modifications of GQDs-based nanocomposites

GQDs, as a component of the graphene family, share a structural foundation with sheets of atoms bonded together in a honeycomb-like configuration. These nanostructures manifest a tunable bandgap, a result of quantum confinement and the edge effect. Their synthesis involves diverse techniques to ensure properties indicates low cytotoxicity, cell membrane permeability, photostability, and resistance to photobleaching are optimized for subsequent biomedical exploration [38, 39]. Various synthetic methodologies contribute to GQDs formation, predominantly employing top-down and bottom-up approaches. Top-down strategies encompass hydrothermal, solvothermal, micromechanical cleavage, thermal reduction, and electrochemical oxidation, each influencing the GQDs properties. Conversely, bottom-up techniques, notably chemical vapor deposition and carbonization methods, involve the drying and carbonization of suitable small molecules or polymers, stepwise organic synthesis, and dynamic functional group acquisition. These intricate procedures enhance the suitability of GQDs for diverse biomedical applications [40–43]. For a comprehensive overview, Table 2 presents a summary of synthesis with surface modification methods applied to GQDs-based nanocomposites, detailing precursor materials, binding energy, and potential applications derived from each method. This synthesis versatility underscores the multifaceted nature of GQDs and their potential in tailored biomedical applications.

**Table 2:** Recent examples of GQDs-based nanocomposites in biomedical applications

Nanocomposite	Synthesis methods	Binding energy	Applications	References
GQDs-FA-EVO	Self-assembly method	Theoretical molecular dynamic calculation	Malignant tumor eradication	[44]
GQDs-ConA@ Fe_3_O_4_	Co-precipitation, pyrolysis	π–π stacking	Cancer cell detection & drug delivery	[45]
N-GQDs/TiO_2_	Hydrothermal	Interfacial charge transfer process	PDT	[46]
FAPEG-TNGs	Hydrothermal method	π-π & electrostatic interaction	Targeted tumor therapy	[47]
PB@PDA@GQDs	Chemical method	_	PPT	[48]
GQDs@ZrO_2_	Hydrothermal method	π–π stacking	Immuno-sensing	[49]
UCNP-GQDs	Hydrothermal method	Covalent bond between GQDs and UCNP	Mitochondrial sensing with PDT	[50]
HMNS/SiO_2_/GQDs	Bottom-up approach	π–π stacking, amino bond, electrostatic interaction	chemotherapy, PTT, PDT	[51]
DOX-MMSN/GQDs	Template encapsulation, solvothermal	Amino bond, electrostatic interaction	Hyperthermia, PTT, chemotherapy	[52]

FA–Folic acid, EVO–Evodiamine, ConA –Concanavalin A, Fe_3_O_4_–Ferric ferrous oxide, N-GQDs–Nitrogen, TiO_2_–Titanium dioxide, PEG–Polyethylene glycol, PB- Prussian blue, PDA- Dopamine hydrochloride, ZrO_2_– Zirconia, UPNP- Upconversion nanoparticles, TRITC Tetramethylrhodamin, DOX- Doxorubicin, MMSN- Mesoporous silica nanoparticles, HMNS- Hollow mesoporous nanoparticles, SiO_2_- Silicon dioxide.

### Surface modification of GQDs and nanocomposite synthesis

GQDs possess the capability to undergo functionalization through various methods, enabling the modification of their properties to suit specific applications. This versatility enhances their potential utilisation across multiple sectors, thereby expanding their range of practical applications. Certainly, there are various restrictions pertaining to the utilisation of pristine and uncontaminated GQDs. Novel functionalities for GQDs can be generated through various methods such as doping, size/shape alteration, and composites formation, thereby enabling the development of new applications. The electrical, optical, and chemical properties of GQDs can be modified by means of functionalization, thereby enabling a diverse range of applications for these materials. The ultimate structure of the GQDs will be predominantly influenced by the intended application of these materials [53–55]. Various types of modifications can be employed, including the two covalent or non-covalent. The decrease in conductivity can be attributed to the alteration of hybridization from Sp^2^ to Sp^3^ in the atom, which occurs because of covalent modification. This modification reduces the influence of the orbital responsible for electronic conjugation [56, 57]. In turn, the non-covalent alteration maintains the extended π conjugation, preserving the GQDs' structural and electrical characteristics. Noncovalent modifications include polymer wrapping, bonding of hydrogen, van der Waals forces, π–π interactions, ionic bonding, and electrostatic effects [56, 58].

The process of amino functionalization, employing primary amine molecules, significantly enhances the electronic properties of N-GQDs. This modification induces considerable electron donation, thereby augmenting the singlet–triplet splitting within amino-functionalized N-GQDs (amino-N-GQDs), consequently boosting the efficiency of intersystem crossing. As a result, the fluorescence emissions of amino-N-GQDs are expected to intensify. Recent investigations have highlighted the robust emission spectra of amino-N-GQDs, contrasting with the weak emission observed in nanocomposites of AgNPs and PEI N-doped GQDs. This enhancement is attributed to the surface conjugation's ability to impede the nonradiative recombination of localized hole-electron pairs into Sp^2^ clusters, consequently improving the surface integrity of the π-electron network and enhancing both electronic and optical characteristics. [59–61]

### Nanocomposite formation from hydrothermal GQDs synthesis and surface modifications

**Fig. 4:**
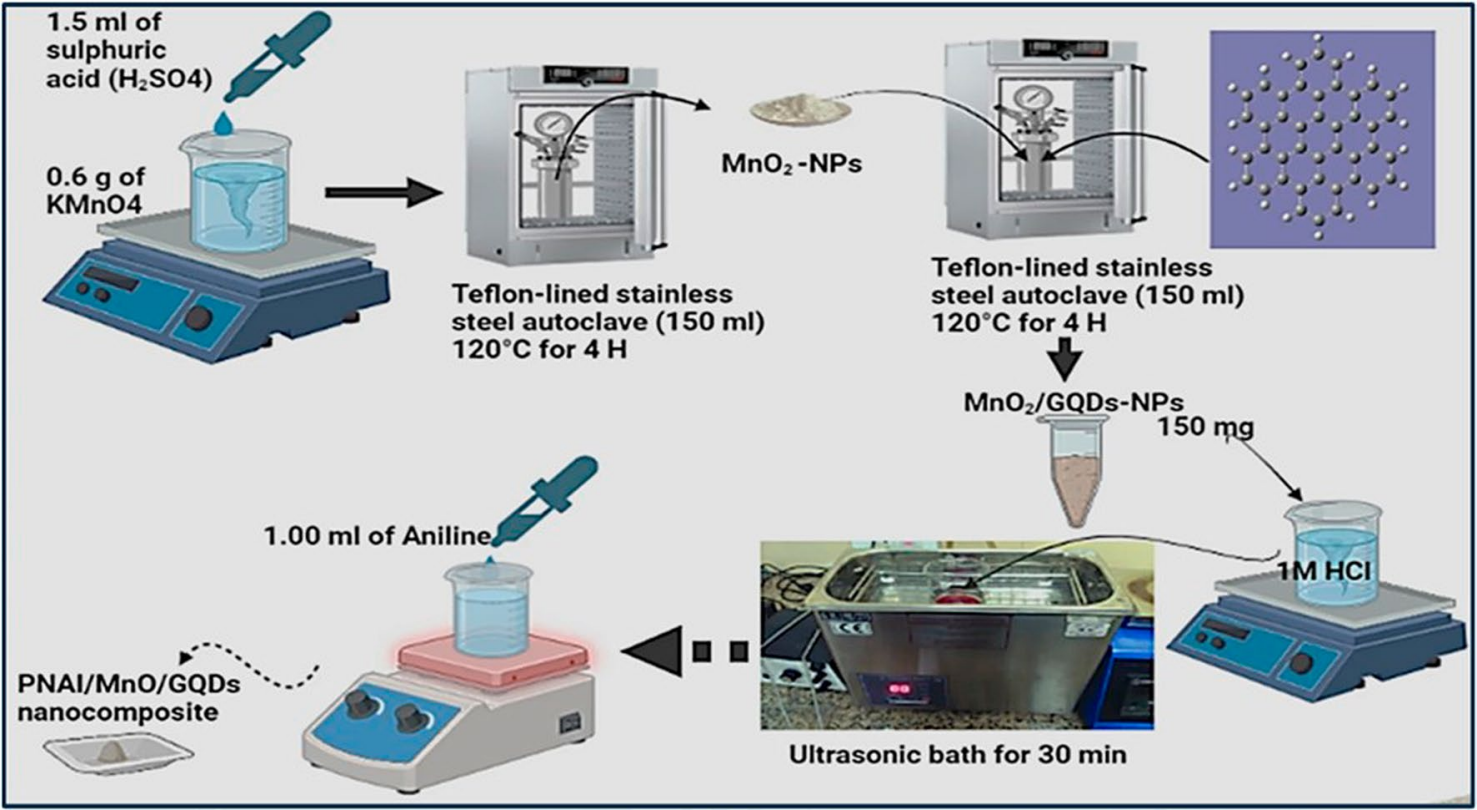
Schematic showing synthesis procedure of GQDs nanocomposite through a hydrothermal process. Reprinted (adapted) with permission from ref. [67] permission conveyed through Copyright 2023, The Author Springer nature

The hydrothermal synthesis method significantly influences both the formation and particle size of GQDs, wherein carbon precursors such as graphite, graphene sheets, and carbon nanotubes undergo oxidation and subsequent treatment at elevated temperatures to yield GQDs. A study by S. Moghimian et al*.,* showcased the production of a GQDs-MoS_2_ (Molybdenum disulfide) nanocomposite thin film through one-step hydrothermal process. The surface chemistry of the MoS_2_-GQDs nanocomposite revealed a surface composition consisting of basal planes adorned with interwoven nanosheets, displaying an average thickness of 15 nm. The nanocomposite holds promise for diverse applications, potentially undercovers new avenues for advanced materials and biomedical technologies [62]. Another group of researchers developed a fluorescent detector using MoS_2_ nanosheets and GQDs to identify epithelial cell adhesion molecules (EpCAM). MoS_2_ nanosheets switched down GQDs fluorescence in this detector utilizing fluorescence resonance energy transfer (FRET). FRET acceptors were MoS_2_ nanosheets and donors were GQDs. This innovative sensor demonstrated promising capabilities for precise and efficient detection of EpCAM, highlighting its potential for biomedical applications [63]. Ramachandran et al*.,* followed the hydrothermal route in which they used citric acid and ethylenediamine concurrently in a magnetically agitated solution for 30 min, followed by a 4-h hydrothermal treatment at 180 °C to produce N-GQDs-TiO_2_ composite. The resulting precipitate was cleaned, centrifuged, and overnight vacuum-dried at 60 °C [46]. In another study conducted by Kumawat M and co-workers, L-glutathione was used as a carbon precursor in a hydrothermal process to create graphene oxide- polyethyleneimine GQDs (GO-PEI-GQDs) that facilitates the electrostatic force at 150 degrees which attract negatively charged GO sheets to positively charged surfaces. The stabilising attachment of the GQDs anionic through the layer-by-layer assembly is made possible by the attachment of the PEI polymeric layer cationic into the GO sheets anionic surface, which results in net charge inversion of the resulting positively charged GO-PEI sheets [64]. Recent approaches to overcome these issues have already been made, with the goal of creating GQDs in a simple, eco-friendly, and controlled manner in one step by modifying the control parameters [65, 66]. Figure 4 depicts the synthesis methodology employed to produce GQDs composite via a hydrothermal process.

### Nanocomposite formation from green GQDs synthesis and surface modifications

Green synthesis represents an innovative approach aimed at minimizing or eliminating the use of hazardous materials in the development, production, and application of chemical products. An example of this approach involves the utilization of biowastes as a cost-effective and sustainable carbon source for GQDs synthesis [68]. Roy P and colleagues pyrolyzed rice husks at 700 °C for two hours to make wrinkled hybrid silicon nanosheets (Si NSs). In addition, GQDs were hydrothermally synthesised from fenugreek seed extracts at 300 °C for eight hours, showing outstanding catalytic activity in oxygen reduction processes. Si-GQDs nanocomposite were produced at three concentrations by stirring 100, 75, and 50 mg of Si NSs with 50, 75, and 100 mg of GQDs in ethanol for 2 h at ambient temperature. The Si-GQD nanocomposite powder was obtained by drying the combined solution overnight at 100 °C [69]. Tajiki et al. reported another instance of green synthesis nanocomposite fabricated by a one-pot photochemical process under an ultraviolet lamp. The procedure entailed the integration of rGO, NGQDs, and metal nanoparticles (AgPd). NGQDs functioned both as a directing and reducing agent, ultimately stabilizing on the surface of rGO through π-π stacking interactions. In this process, the nitrite oxidation reaction was electro-catalyzed using silver and palladium nanoparticles. Cyclic voltammetry data indicated that the nitrite oxidation mechanism using this technique was diffusion-regulated. Furthermore, the constructed sensor's sensitivity and detection limit were determined through chronoamperometry experiments, revealing superior performance compared to most recently developed sensors [70]. In a recent investigation, Teymourinia et al. created GQDs nanocomposite solvothermally using corn powder. The resulting trisulfide/GQDs were then dispersed into a 30 mL solution with a concentration of 0.045 M and sonicated for 15 min. A mixture of 5 mL of titanium tetraisopropoxide and ethanol at a 1:5 ratio was prepared and added dropwise to the solution under sonication. The mixture was stirred vigorously overnight. Additionally, the nanocomposite shows very high antibacterial activity [71]. These instances underscore the multifaceted applications of green synthesis in producing GQDs with diverse properties, showcasing its potential in environmentally friendly and economically viable approaches to advanced sensor technologies and other applications in the biomedical domain.

### Nanocomposite formation from pyrolytic GQDs synthesis and surface modifications

**Fig. 5:**
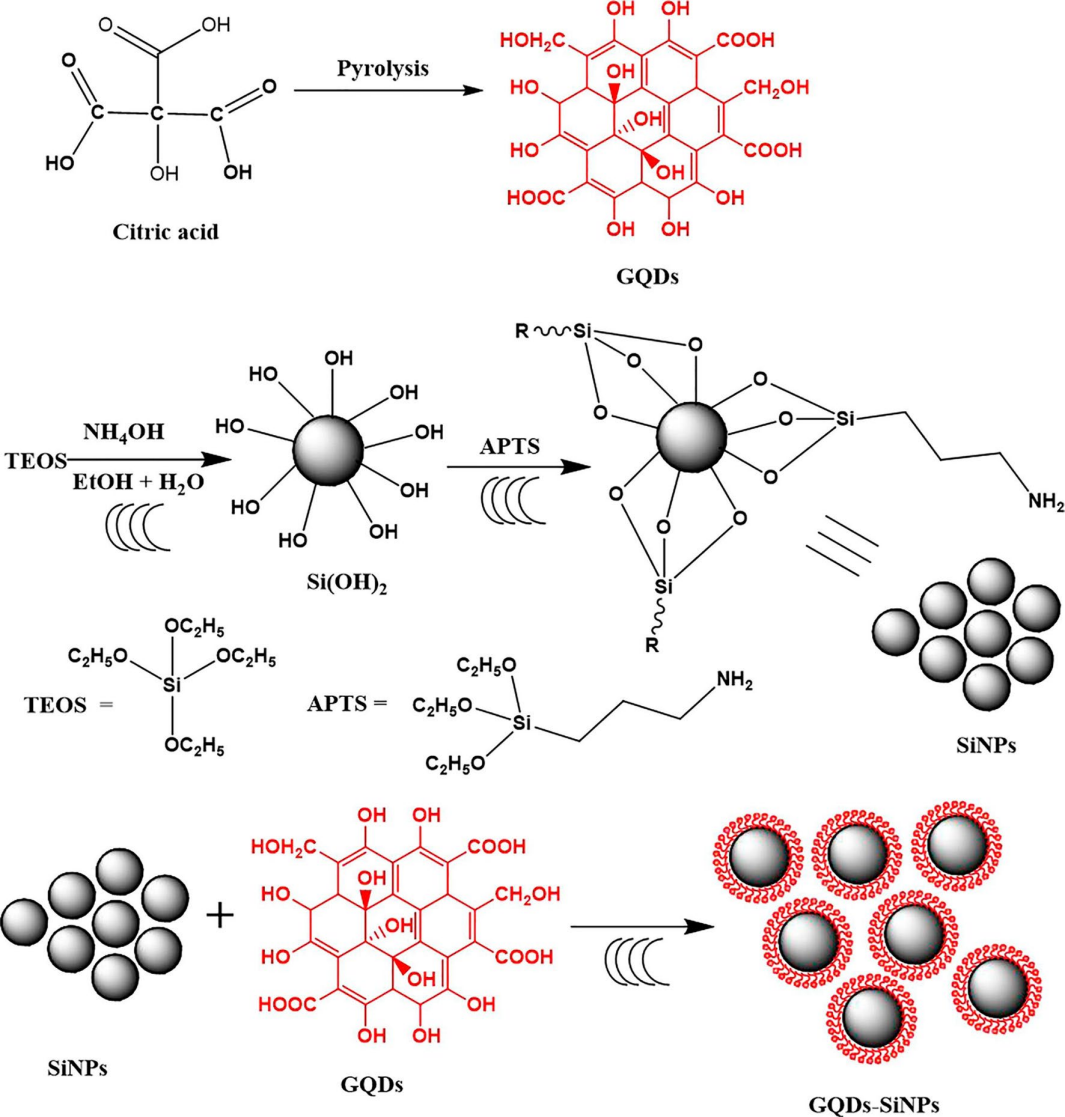
Schematic illustration of the surface modified GQDs nanocomposite synthesis via pyrolysis procedure with ultrasonic assistance. Reprinted (adapted) with permission from ref. [77] Copyright (2021) Published by Elsevier

Pyrolysis is the chemical breakdown of organic compounds such as citric acid, acetylacetone, dioxins, and polycyclic aromatic hydrocarbons triggered by heat in the absence of oxygen [72, 73]. In a study performed by Kadian. S and colleagues fabricated a nanocomposite of silver nanoparticles (AgNPs) coated with sulfur-doped GQDs (S-GQDs) using pyrolysis and HEK 293 cell lines were used to study the cytotoxicity of the nanocomposite. Surprisingly, compared to AgNPs and S-GQDs, the nanocomposite demonstrated improved cell survivability and offers potential as an industrial antibacterial agent [74]. Rahimi K and colleagues employed a straightforward pyrolysis technique for the isolation of zinc oxide nanorods (ZnO NRs) and GQDs. Subsequently, the structures were amalgamated, and it was demonstrated that they exhibited superior catalytic efficacy in comparison to the constituent materials. The encapsulation of ZnO nanorods by GQDs resulted in a decrease in the bandgap from 3.2 to 2.8 eV [75]. Similarly, Alvand and colleagues successfully synthesized a multifunctional nanocomposite consisting of iron oxide/silcon/GQDs (Fe_3_O_4_@SiO_2_@GQDs) with an average diameter of approximately 22 nm. Through surface modification, the amino-functionalized Fe_3_O_4_@SiO_2_ nanospheres were affixed to a mixture comprising the prepared GQDs and EDC, facilitating their dispersion. Fluorescent probes are employed for the purpose of detecting and eliminating Hg^2+^ from polluted water sources [76]. Nanocomposites of GQDs have also been fabricated using other techniques such as simple stirring, electrochemical oxidation, microwave method, calcination, and by utilizing natural products such as rice husk and corn powder [26]. Figure 5 shows the fabrication of a nanocomposite using GQDs and amine-functionalized silica nanoparticles. To increase surface area, SiNPs are functionalized with(3-Aminopropyl) triethoxysilane (APTES). GQDs are ultrasonically combined with amine functionalized SiNPs to make the composite.

## Classification of GQDs-based nanocomposites

GQDs-based nanocomposites are systematically formulated by incorporating various nanomaterials to cater to diverse applications in the biomedical sector. Despite the exceptional qualities of GQDs, their standalone application may present drawbacks related to conductivity, toxicity, and quantum yield [22, 78]. To address these limitations and optimize properties for a range of scientific applications, GQDs are often combined with other materials, resulting in nanocomposites. The upcoming section will delve into GQDs-based nanocomposites that integrate polymer, liposomal, magnetic, and metallic materials. This strategic integration enhances the overall performance and functionality of GQDs, expanding their utility in biomedical applications. The synergistic effects achieved through these nanocomposites offer tailored solutions to overcome the inherent limitations of GQDs when used independently.

### GQDs-based polymer-nanocomposites

An important advancement in the realm of nanomedicine is the creation of polymeric nanocomposite based on GQDs. The most prevalent organic materials for hosting GQDs are polymeric structures. In these materials, GQDs not only respond to outside stimuli with photoluminescence but also function as nano-catalysts to increase the durability of the nanocomposite through the development of either covalent or non-covalent interactions between the secondary chains and surface functional groups of the GQDs and polymer matrices [79]. Numerous polymers, such as polyethylene, cellulose, starch, polydopamine, polyaniline, polyethylenedioxythiophene, polyvinyl-alcohol etc., are combined for developing polymeric nanocomposite with GQDs [80–82]. Because of their highly unique surface characteristics, aggregation, and hydrophobic relations, GQDs functionalized with different polymers exhibit improved biocompatibility, physiological stability, and reduced toxicity and are crucial for biomedical applications such as drug delivery, cell culture, and tissue engineering, antibacterial and anti-diabetic potentials, biosensing, and bioimaging [83]. 

A variety of techniques have been employed for the preparation of polymer GQDs composites, such as non-covalent dispersion methods, solution intercalation, in situ polymerization, melt intercalation, in situ mini-emulsion polymerization, etc [84–86]. In a study conducted by Gebreegziabher et al. they used the chemical in situ polymerization approach and created polyaniline (PANI)-GQDs hybrid nanocomposite, and found that it had better thermal stability and conductivity using the Thermogravimetric analysis (TGA) and instrumental variable (I–V) analyses, respectively [87]. In another work done by Chen W and co-workers, they employed a facial synthesis approach with starch as a precursor and successfully used GQDs composite for cell imaging because of its strong PL emission, low cytotoxicity, and good hydrophilicity [88].

Yang C and his group synthesized biodegradable charged polyester vectors (BCPVs) with GQDs for pancreatic cancer therapy applications. The resultant nanocomposite has shown outstanding K-ras downregulation activity, high stability in physiologically realistic environments, and efficient bioactivity suppression of pancreatic cancer. More crucially, NIR laser light was used to destroy the cells by heating up the composites via the photothermal phenomenon. This heat then caused the nanocomposite to release its contents. The anticancer activity of the nano-complexes was greatly improved by the subsequent triggered release function [89]. Additionally, in recent work on colon cancer suppression analysis, Lee G. and his associates used PEI conjugated GQDs and a type of green fluorescent protein, to test the drug carrier's delivery capabilities. Intrinsic—π–π interaction was used to encapsulate DOX and create GQDs-polymer-DOX conjugates. The in vivo and in vitro data suggest that the nanocomposite has a great ability for tumour suppression and may be used in the treatment of cancer in the future. [90]

### GQDs-based metallic nanocomposites

**Fig. 6:**
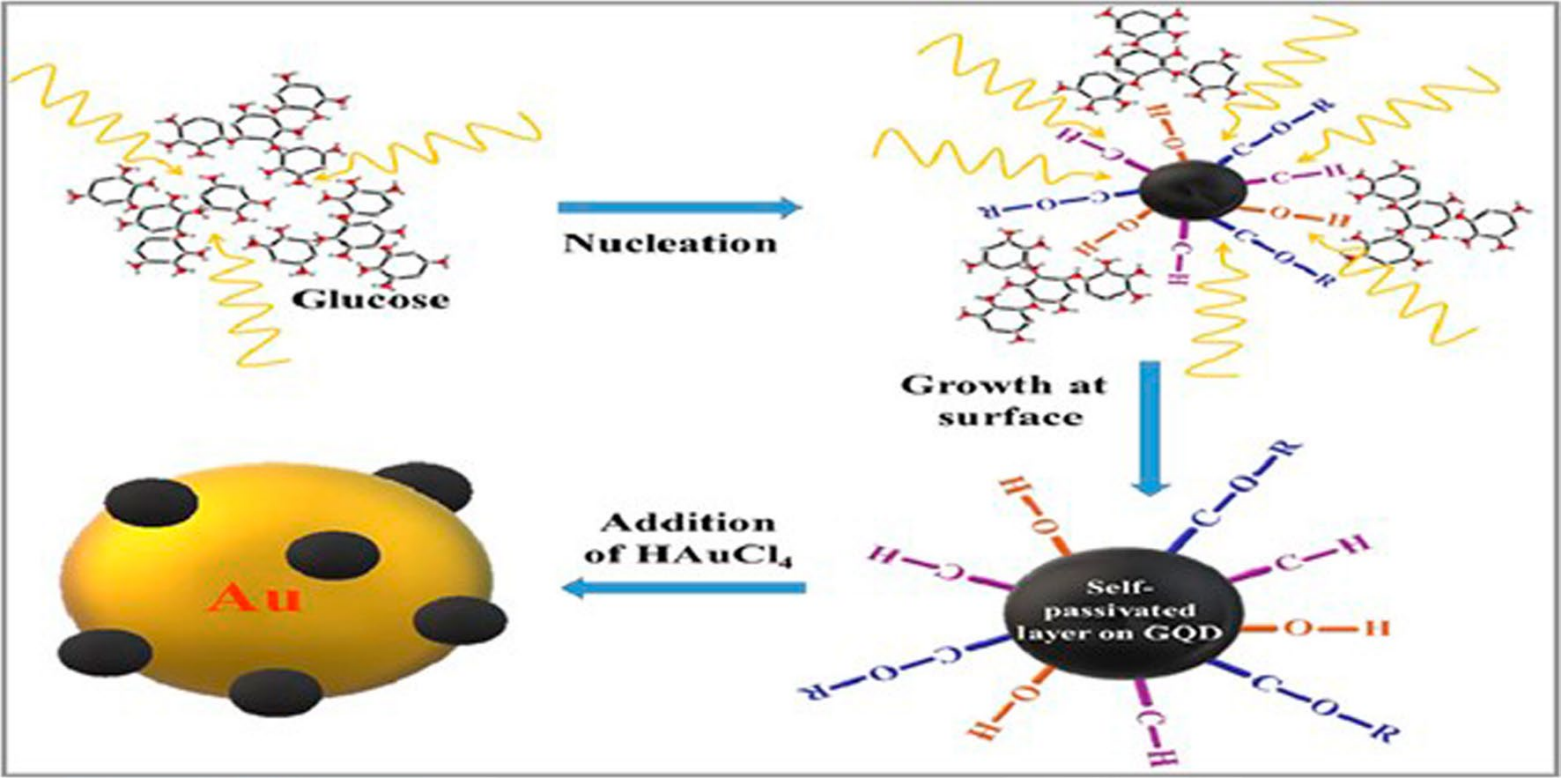
Schematic representation depicting the interaction of GQDs with AuNPs to form hybrid nanocomposite. Reprinted (adapted) with permission from ref. [97] Copyright (2020) Elsevier

The most promising materials in nanotechnology are carbon allotropes and metallic nanoparticles. [91] In general, in-situ and ex-situ preparations can be used to produce GQDs nanocomposites containing inorganic compounds. The choice of an appropriate strategy can improve nanofabrication because each process has benefits and limitations of its own. Various metallic nanoparticles such as gold (Au), bismuth (Bi), silver (Ag), cobalt (Co), platinum (Pt), magnesium (Mg), and cadmium are utilised to create GQDs-based metallic nanocomposites [92, 93]. For instance, Ganganboina et al. first produced Au-PANI nanocomposites by oxidising aniline interfacially in the presence of Au^3+^ ions in water and toluene. The hydrothermal technique was used to create the N, S-GQDs. To confirm that N, S-GQDs were linked to Au-PANI via the Au-thiol interaction to generate N, S-GQDs@Au-PANI nanocomposites, N, S-GQDs and Au-PANI were then combined and stirred for 24 hours for detection of carcinoembryonic antigen. It is determined that the lattice fringe of the N, S-GQDs on the Au-PANI nanowires, which was visible in TEM pictures, is 0.21 nm, which is consistent with the (100) plane of GQDs as-prepared [94]. Similarly, Jin et al. reported the creation of nanocomposites for surface-enhanced raman scattering (SERS) and catalytic treatment specifically targeted towards tumours by hydrothermally processing a mixture of GQDs and AgNO_3_ for 30 minutes at 120 °C. The authors continued by explaining that the core and shell of their core-shell nanostructured (10nm) nanocomposites were made of AgNP and GQDs, respectively [95]. In a distinct work, reported by Liu J his team prepared (AuNPs) /glycine(G_n_)/GQDs nanocomposite. In summary, 100μl of AuNPs aqueous solution received 3ml G_n_ at various concentrations along with 100μl of GQDs using the hydrothermal process. According to transmission electron microscopy, AuNPs are monodispersed and spherical and the size of AuNPs ranges from 45 to 60 nm. The interaction between AuNPs and GQDs can be changed by altering the chain length of G_n_. In addition, the AuNPs/G_n_/GQDs combination showed excellent fluorescence in cells and low toxicity [96]. Fig. 6 illustrates the process of generating GQDs nanocomposite using metallic materials.

### GQDs-based magnetic nanocomposites

Numerous uses for magnetic nanoparticles have been discovered in the fields of bioimaging, magnetic hyperthermia therapy, drug delivery, and nanomedicine. Fe_3_O_4_ nanoparticles seem an ideal choice for a GQDs-based nanocomposite because they are less toxic, superparamagnetic, environmentally friendly, and biocompatible than other magnetic nanoparticles [98]. According to our understanding, the GQDs nanocomposite with Fe_3_O_4_ have ample opportunity for exploration and advancement. Although there are few articles available with promising results in biomedical applications. For example, Chen et al. created a GQDs/magnetic chitosan formulation in which DOX was incorporated into chitosan via the aldehyde group, ferrous nanoparticles were formed by co-precipitation, and GQDs were altered on the surface of magnetic chitosan via an amide bonding. The drug delivery nanosystems treat tumours with synergistic effects, according to the investigation conducted *in vivo* and *in vitro*, nanocomposite featuring a promising platform for the treatment of hepatocellular carcinoma [99]. 

PEG was used in similar research by Bernad S and colleagues to improve the stability and biocompatibility of nanocomposite. TEM images show that a light GQDs background surrounds the spherical magnetic NPs. The drug release tests showed a pH-dependent profile, with higher rates of release observed at an acidic pH of 5.0 relative to a neutral pH of 7.4 [100]. In another research study published by Hasanzadeh the researchers created GQDs using pyrolysis and the formation of nanocomposites using the one-step co-precipitation method by vigorously stirred Fe^3+/^GQDs under nitrogen flow. To confirm their morphology characterisation techniques were used and concluded that nanocomposite has superior performance for electrochemical oxidation of amino acids and detection, respectively [101].

### GQDs-based liposomal nanocomposites

**Fig. 7:**
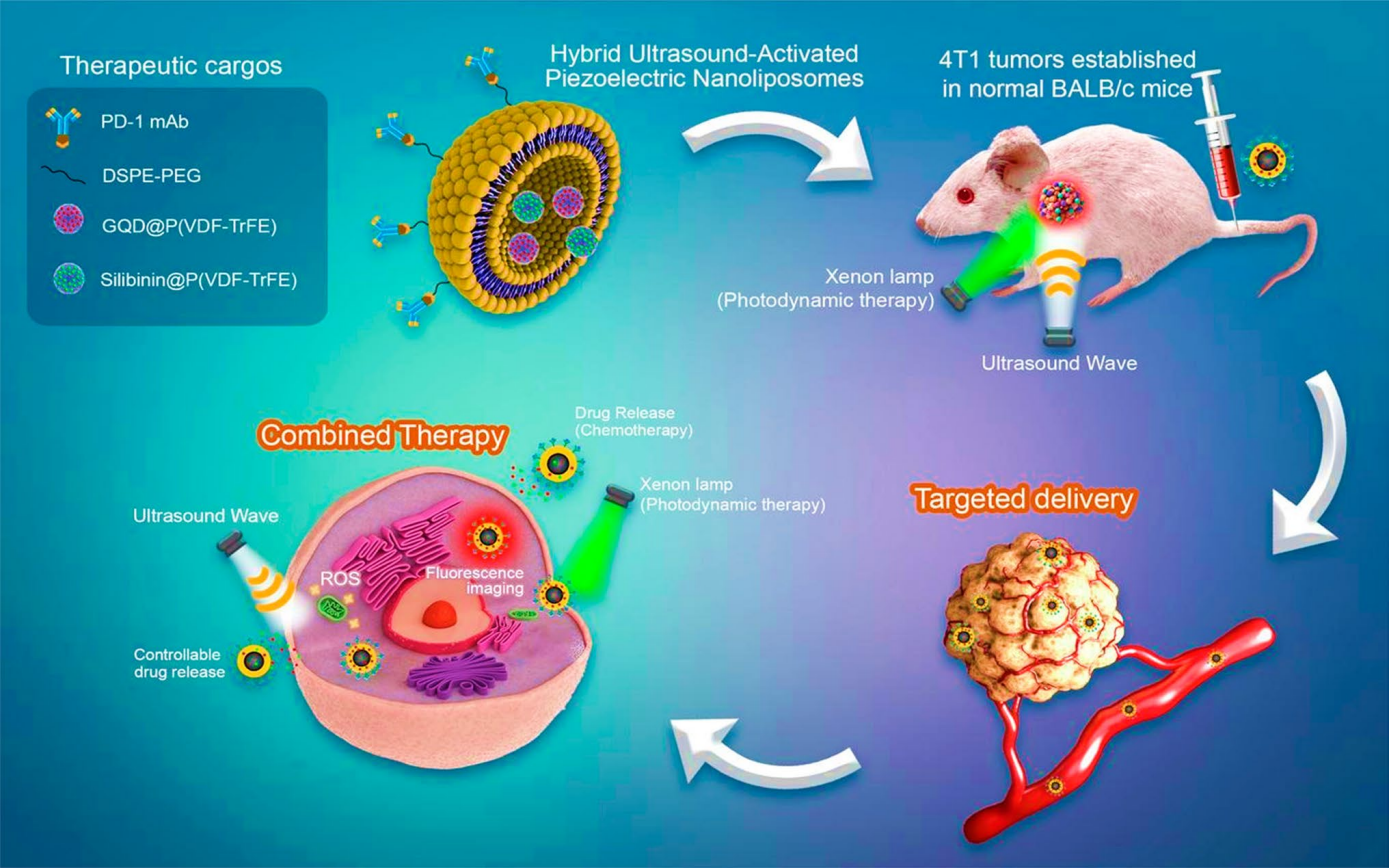
Graphical illustration of the efficient liposomes with dual-stimuli response nature for cancer treatment and bioimaging. Reprinted (adapted) with permission from ref [104]. Copyright 2022, International Journal of Pharmaceutics

Liposomes are spherical membranes made of a phospholipid bilayer that replicates the fluid mosaic structure and characteristics of the cell membrane. They are hydrophilic and hydrophobic environments [102]. In a recent study, Liu C. and colleagues achieved a high loading of GQDs with the size of (4 nm) into the aqueous core of liposomes (45.68 ± 1.44%) which was regulated by pressure. They also demonstrated good photothermal therapeutic effectiveness in vitro, killing about 75% of cancer cells and proving to be an effective tool for quick renal clearance [103]. In another study conducted by Ramedani *et.al.,*hybrid liposome nanocarrier that includes a piezoelectric polymer, GQDs and Silibinin were fabricated by the hydration thin-film method, with an average size of (230 ± 20 nm). The researchers demonstrated how the created hybrid nanoparticles allow for simultaneous fluorescence imaging of cancer cells in vivo and piezoelectric-stimulated drug administration [104]. Moreover, modified AuNPs and GQDs were added to a phospholipid thin film during the film hydration process to create a multifunctional liposome by Prasad R and his team [105]. DOX was also encapsulated on a nanocomposite to demonstrate photo-triggered chemotherapy and cell targeting. The composite uncovered new possibilities for targeted imaging and cancer therapy by demonstrating the bimodality for in vivo tumour diagnostics and tumour reduction. The visual representation in Fig. 7 depicts the composite of GQDs liposomes, which demonstrates a responsive characteristic to dual stimuli. This property makes them well-suited for applications in cancer therapy and bioimaging.

## GQDs-based nanocomposites in vitro and in vivo investigations

Despite being in the early stages of research, GQDs have already exhibited acknowledged biocompatibility and low cytotoxicity in both in vitro and in vivo studies. These findings have sparked increased interest in GQDs as nanomaterials, surpassing the appeal of other quantum dots [83, 106]. The toxicity studies conducted on GQDs based nanomaterial have yielded encouraging findings. Dutta Chowdhury et al. demonstrated that DOX-loaded nanocarriers GQD-ConA@Fe_3_O_4_ were cultured in HeLa and endothelial cells for 12 hours in the presence of a magnetic field. The data revealed that these nanocarriers had no discernible effect on cell viability, which remained above 90%. The findings also revealed a highly targeted intracellular release of DOX within cancer cells. [45] In another work by Ramachandran et al. nanocomposite dosages (0.1 and 0.5 mg mL^−1^) caused MDA-MB-231 cells to perish more than HS27 cells. Cancer cells had enhanced reactive oxygen species (ROS), particularly singlet oxygen. Cancer cell death pathways were investigated using Caspase Glo-3/7.

**Fig. 8:**
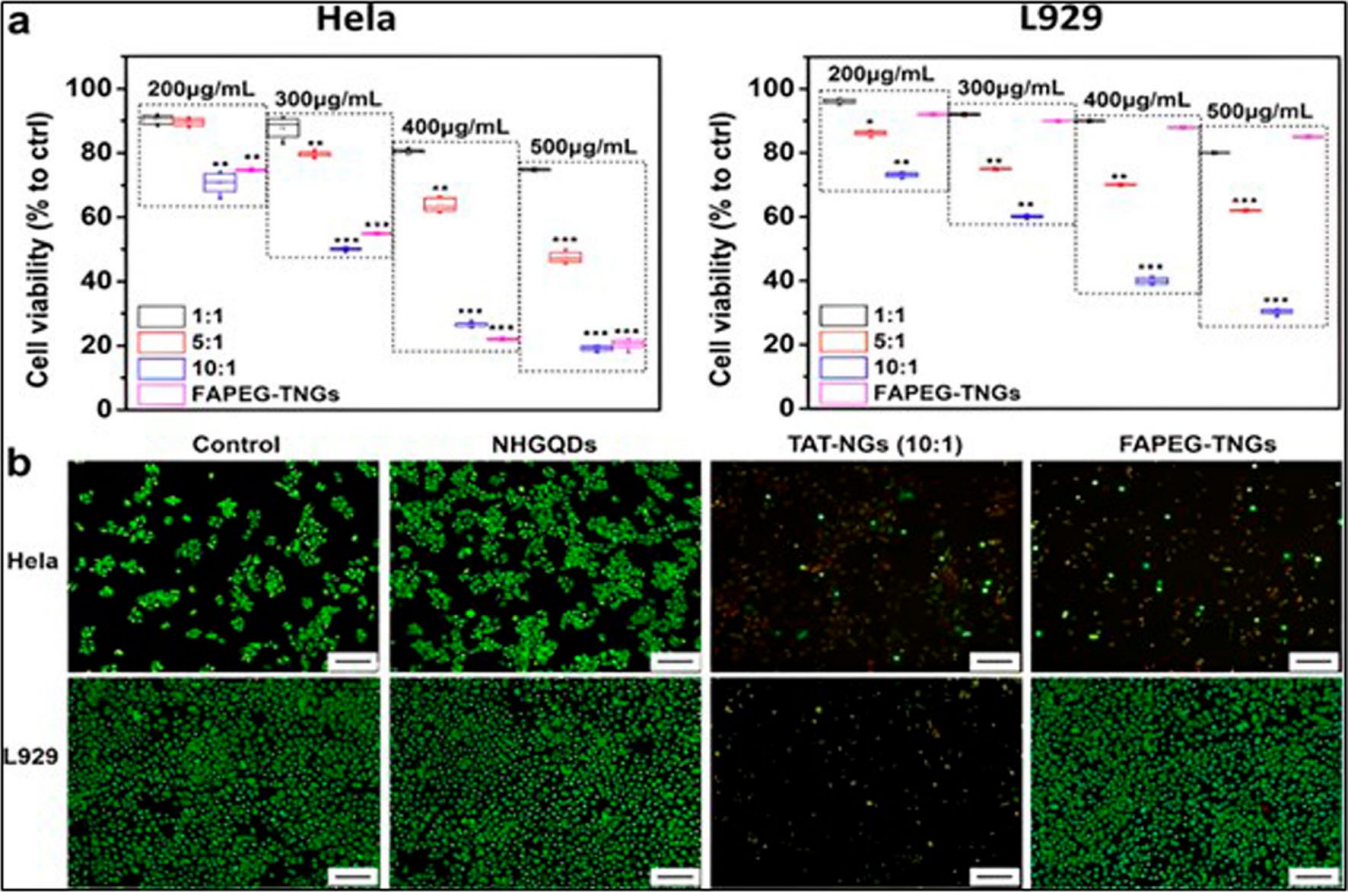
TAT-NGs and FAPEG-TNGs' 24-h effects on HeLa or L929 cell survival. **a** CCK-8 test, **b** Calcein-AM/PI live/dead staining. Reprinted (adapted) with permission from ref [108]. (2021) Springer Nature

The nanocomposite could induce mitochondrial-dependent death in targeted cells as a photosensitizer [46]. Chen and co-workers examined the biological toxicity and side effects of altering the size of the synthesised material GQDs/magnetic chitosan and PTT treatment intensity in hepatocellular carcinoma (HCC). Their study found no in vivo or in vitro toxicity or harmful consequences. Due to its tumour cell targeting, the DOX-Fe_3_O_4_@CGA group outperformed DOX in tumour cell elimination [107]. Figure 8 depicts the vitality of HeLa and L929 cells. The amine-functionalized GQDs were modified by the addition of a nucleus targeting TAT peptides (TAT-NGs). TAT-NGs were then further changed by including cancer-cell-targeting folic acid (FA) modified PEG via a disulfide bond, yielding folic acid modified PEG via disulfide linkage (FAPEG-TNGs). Furthermore, most of the NH-GQDs-treated HeLa and L929 cells were green-stained and alive, demonstrating that the NH-GQDs are having cytocompatibility.

Prasad, Rajendra et al. examined liposomal nanotheranostic systems with GQDs and AuNP in vivo. Liposomal nanohybrids scavenged ROS in this investigation. During treatment with near-infrared (NIR) light, the tumor's surroundings were unharmed. In tumour-bearing mice, the nanohybrids were biocompatible, reducing haemolysis, increasing cell viability, maintaining animal health, and controlling body weight. Nanohybrid chemo phototherapy reduced tumours more than single therapy [105]. Similarly, Dong and colleagues created PEG modified GQDs@hMSN-PEG (hollow mesoporous silica nanoparticles), which was a good candidate for drug transport and delivery due to its fluorescence, structural stability, and huge porosity confirmed by vivo organ distribution and histopathology tests. In tumor-bearing animals, DOX distribution to MCF-7 tumours was enhanced. [109] Kersting, David et al. examined GQDs absorption in C57BL/6 mice liver tissue ex vivo*.* Precision-cut tissue slices (PCLS) show more gene expression similarity than liver cell lines and hepatocyte cultures. The liver's role in nanoparticle biotransformation, metabolism, and toxicity makes this finding significant. After cultivation, histological analysis of precision-cut mammary tumour slices (PCMTS) showed that 90% of cells were viable and 10% were necrotic [110]. GQDs' low toxicity and biocompatibility make them promising for numerous applications. However, the toxicity profile of GQDs varied with different investigations, and there is evidence that certain GQDs can kill cells by generating intracellular ROS [111]. Due to the diversity and complexity of GQDs surface changes, a thorough evaluation of their toxicity and function is still missing in experimental and theoretical simulation. Several crucial factors contribute to the mitigation of toxicity, including the regulation of GQDs concentration. Additionally, the toxicity of GQDs is subject to variation based on the specific synthesis process employed. Furthermore, it has been observed that GQDs with nano-meter dimensions pose a lower level of harm when compared to GQDs with micro-meter dimensions. Surface modification and functionalization also improve GQDs characteristics for biomedical applications [112–114]. 

## Biomedical application of GQDs-based nanocomposites

Although GQDs possess remarkable properties, as discussed in the previous sections, their exclusive utilisation may not be sufficient for certain applications. Consequently, the enhancement of performance can be achieved through surface functionalization and the incorporation of supplementary materials. In this scenario, GQDs and other materials were mixed to create hybrid structures with features that were optimised for a variety of technological applications. In particular, the subsequent sections will primarily emphasise biomedical applications involving composites based on GQDs. Table 3 provides a comprehensive overview of various biomedical applications where GQDs have shown promising potential.

**Table 3:** Lists promising clinical applications utilising GQDs

Material	Modification	Application	Results	References
GQDs	Polyurethane	Tissue engineering	GQDs make nanofibers more regular and help them form straighter nanofibers, which suggests that they might be useful for wound healing	[115]
GQDs	zinc oxide (ZnO)	Antimicrobial activity	The antibacterial activity of GQDs is enhanced by their reduced size and better adsorption of the nanocomposites onto bacterial cell surfaces	[116]
GQDs	Gadolinium oxide (Gd_2_O_3_)	MRI contrast agent and cell labelling agent	The modification process of GQDs combines MRI features with both one-photon and two-photon imaging properties, making it a suitable dual-modal imaging agent	[117]
NGQDs	Chitosan	Biomedical Implants	Enhancement of biocompatibility and functionality of bio tissue implants	[118]
GQDs	Poly(l-lactide) PLA and PEG	Gene-Targeting Agents	Functionalized GQDs exhibit excellent biocompatibility, reduced cytotoxicity, and protective characteristics	[119]
GQDs	AgNPs	Cancer theranostics	The synergistic anticancer impact of Ag^+^ and oxidised tannic acid kills cells efficiently, while GQDs are used for cell imaging to assess therapy efficiency	[120]
GQDs	Bovine serum albumin, poly lactic-co-glycolic acid	Drug Delivery and Chemo photothermal/PDT	The pH-responsive multifunctional nanoparticles, synthesised easily, destroy tumour cells and show promise for synergistic therapy	[121]

### Drug delivery

**Fig. 9:**
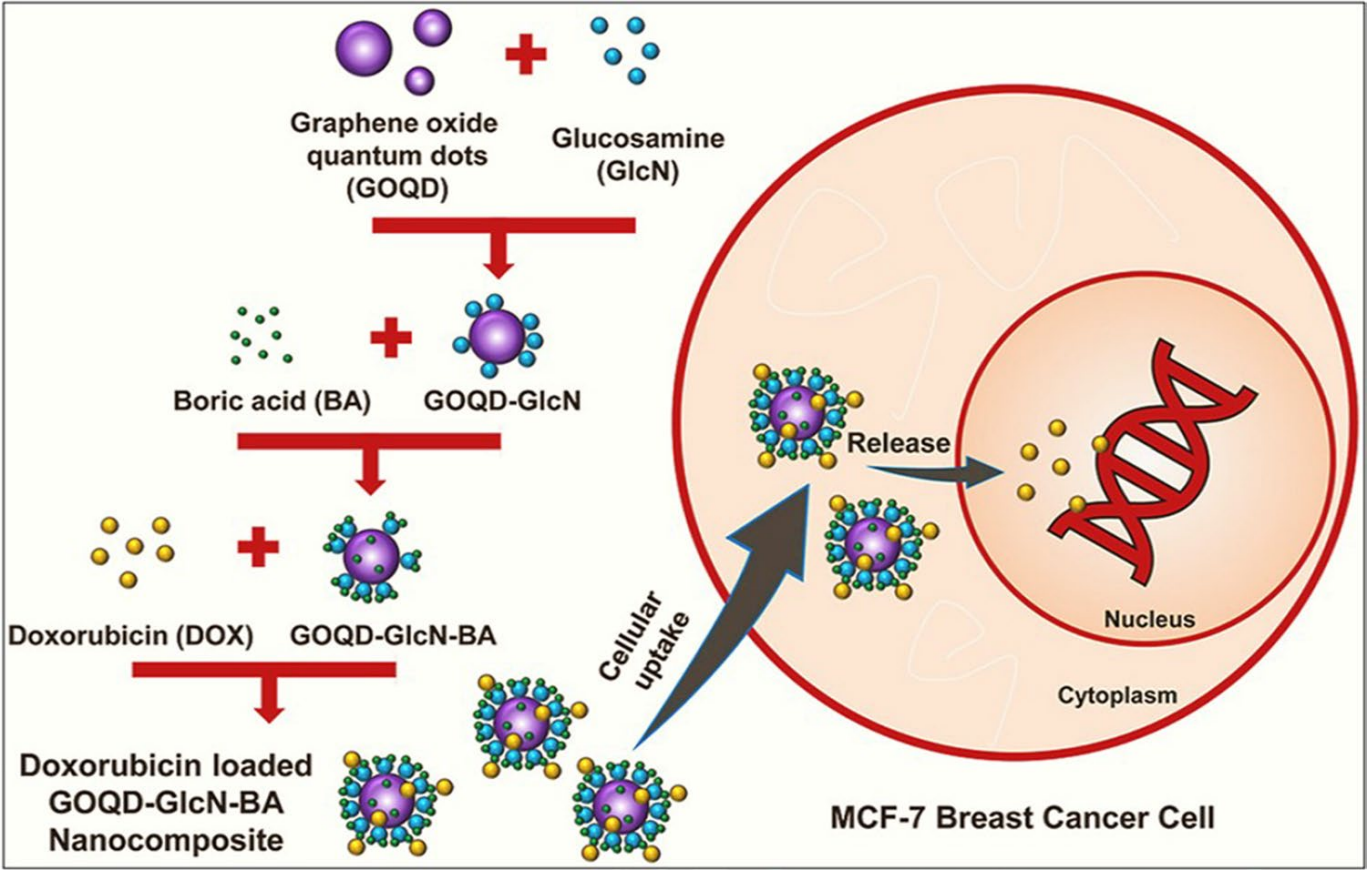
Graphical illustration of the GQDs nanocomposite for cellular uptake and release in breast cancer cells. Reprinted (adapted) with permission from ref. [126] Copyright (2023) Elsevier

Most of the drugs can be loaded quickly because GQD-based nanocomposites can bind to different biological materials via π–π stacking and electrostatic interaction. GQDs exhibit effective drug molecular loading cores because of their higher specific surface area, improved water solubility, and less cytotoxicity [83]. The rapid delivery rate of GQDs, which prolongs the time of blood circulation, is another aspect that enhances their efficacy [122]. Some researchers have theoretically studied the properties of GQDs using molecular dynamics (MD) simulations and density functional theory (DFT) calculations to better use GQDs for drug delivery [123, 124]. The rapid delivery rate of GQDs, which prolongs the time of blood circulation, is another aspect that enhances their efficacy. The therapeutic efficacy of drugs cannot be improved merely by concentrating on drug delivery and disregarding drug release [125]. As a result, there is an increasing need for researchers to comprehend the intimate connection between the delivery of drugs and release, and work on creating various drug delivery-release mechanisms with different materials. Figure 9 depicts a visual representation of the nanocomposite consisting of GQDs utilised for drug delivery purposes.

GQDs may offer more bonding sites for chemotherapeutic conjugation and better cell absorption when paired with other organic and inorganic conventional nanoplatforms [127]. In some investigations, GQDs-based nanocomposite serves as a reliable drug delivery system and fluorescent imaging probe, enabling the tracking of a carrier's intracellular localization and the path that the drug travels after entering the carrier [128, 129]. Recent research also looked at PEG, chitosan, and dextran/poly(N-isopropylacrylamide) (Dex/PNIPAM) as GQDs-based polymers for drug administration. Findings indicate that the synthetic GQDs composite is exceptionally efficient in delivering drugs to cancer cells while also being biocompatible. The same is true for the complicated arrangement of targeted ligand medications in nanomaterials [130, 131]. GQDs represent a promising avenue in drug delivery owing to their multifunctional properties, including high surface area and effective drug loading capabilities. However, while studies demonstrate their potential, there's a need for further investigation into the precise mechanisms of drug release and the optimization of delivery-release strategies. Additionally, exploring the synergistic effects of GQDs with other nanoplatforms could enhance their efficacy in targeted drug delivery systems. It's crucial for future research to address these challenges to unlock the full potential of GQDs in biomedical applications.

### Biosensing

**Fig. 10:**
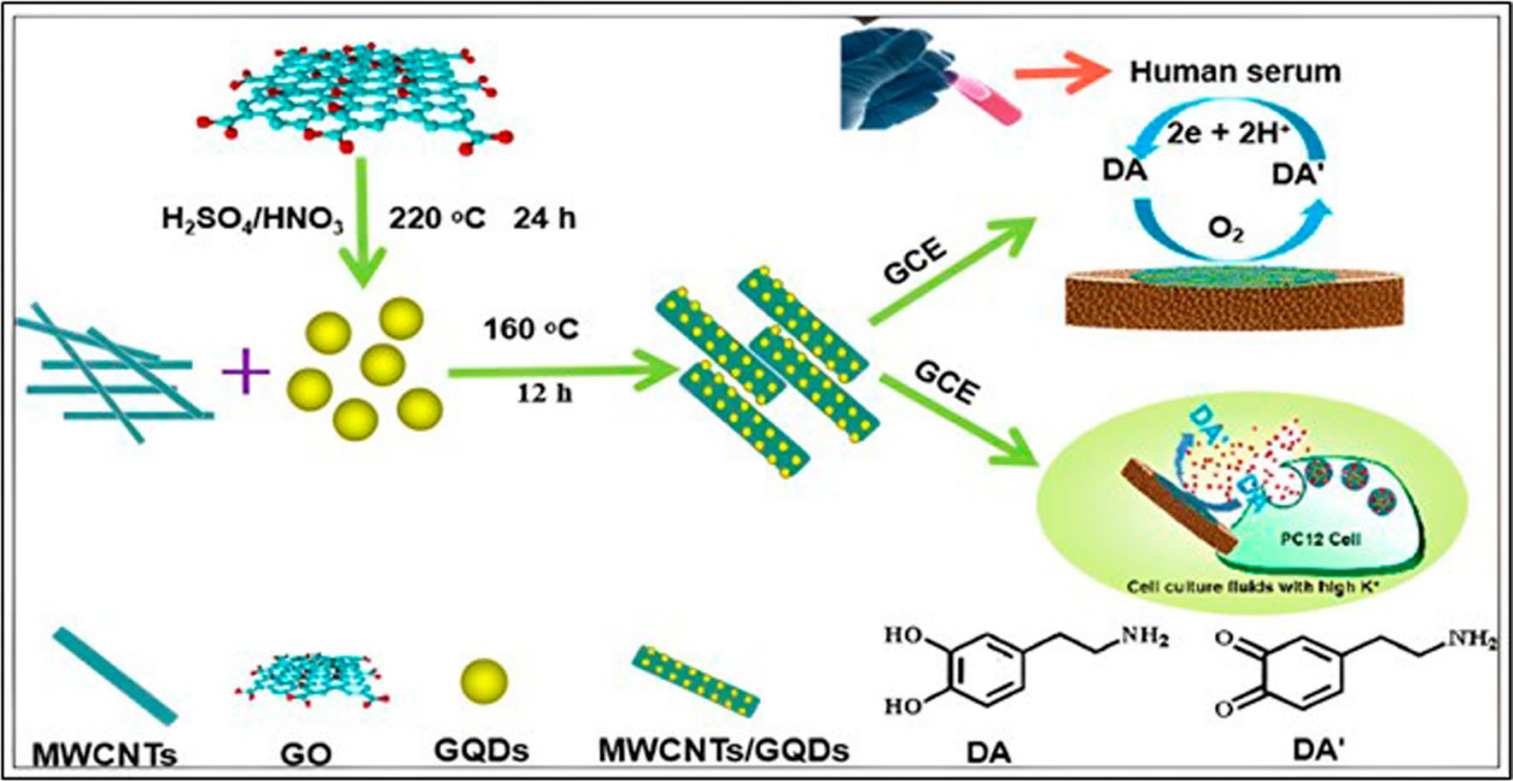
Schematic for an electrochemical dopamine sensor using multiwalled carbon nanotubes (MWCNTs)/GQDs. Reprinted (adapted) with permission from ref. [134] Copyright (2020) American chemical Society

Biosensing platforms provided a wealth of potential for diagnosing a variety of illnesses, including cancer, cardiovascular, viral, and neurological diseases. Early disease detection is essential for eradicating metastases and avoiding fatalities [132]. In this context, the current part will cover recent developments in platforms for electrochemical biosensing, electrochemiluminescence, and fluorescent-based GQDs. In accordance with this, Miao X and his colleagues developed monodisperse N-doping GQDs-wrapped gold nanoparticles for Surface-enhanced Raman spectroscopy (SERS)-based sensing and cell imaging. These nanoparticles exhibit excellent biocompatibility, stability, and excellent enhancement ability [133]. Figure 10 depicts a schematic representation of electrochemical sensing leveraging nanocomposite based GQDs.

Another study reported by Lei Y and co-workers developed solvothermally W_18_O_49_/GQDs (tungsten oxide) composites with exceptional stability and homogeneous particle dispersion. As a result, changing GQDs will be a useful technique to enhance W_18_O_49_'s SERS performance [135]. In a way similar, Sun Y and his team developed a molybdenum disulfide-based GQDs nanocomposite and combined it with enzyme-assisted recycling DNA for sensitive analysis of DNA sequences, offering a powerful approach for biomedical research [136]. Furthermore, Du J and team also generated luminous composite hydrogel poly(AM-co-AA)/GQDs using in situ free radical polymerization, which had remarkable mechanical strength, resistance to fatigue, and natural healing qualities. As a result, the nanocomposite worked well as a sensor to find aqueous Fe^3+^ contamination in water [137]. The current standards for sensing in GQDs involve addressing key challenges such as cost-inefficient synthesis routes, time-consuming processes, and low sensitivity in complex samples. Recent advancements have focused on enhancing sensitivity, specificity, and selectivity by combining GQDs with electrochemical biosensors. Optimizing bioconjugation and biofunctionalization strategies, as well as the interplay between the size and shape of biorecognition elements and GQDs, are crucial for achieving high reliability and sensitivity in GQDs-based electrochemical biosensors. Moreover, there is a need to validate the technology using human samples to ensure accuracy and applicability in clinical settings. Overall, ongoing research aims to develop efficient biosensing platforms capable of real-time and rapid monitoring to improve diagnostic accuracy and patient outcomes in conditions different medical conditions [138–140]. 

### Photothermal therapy

**Fig. 11:**
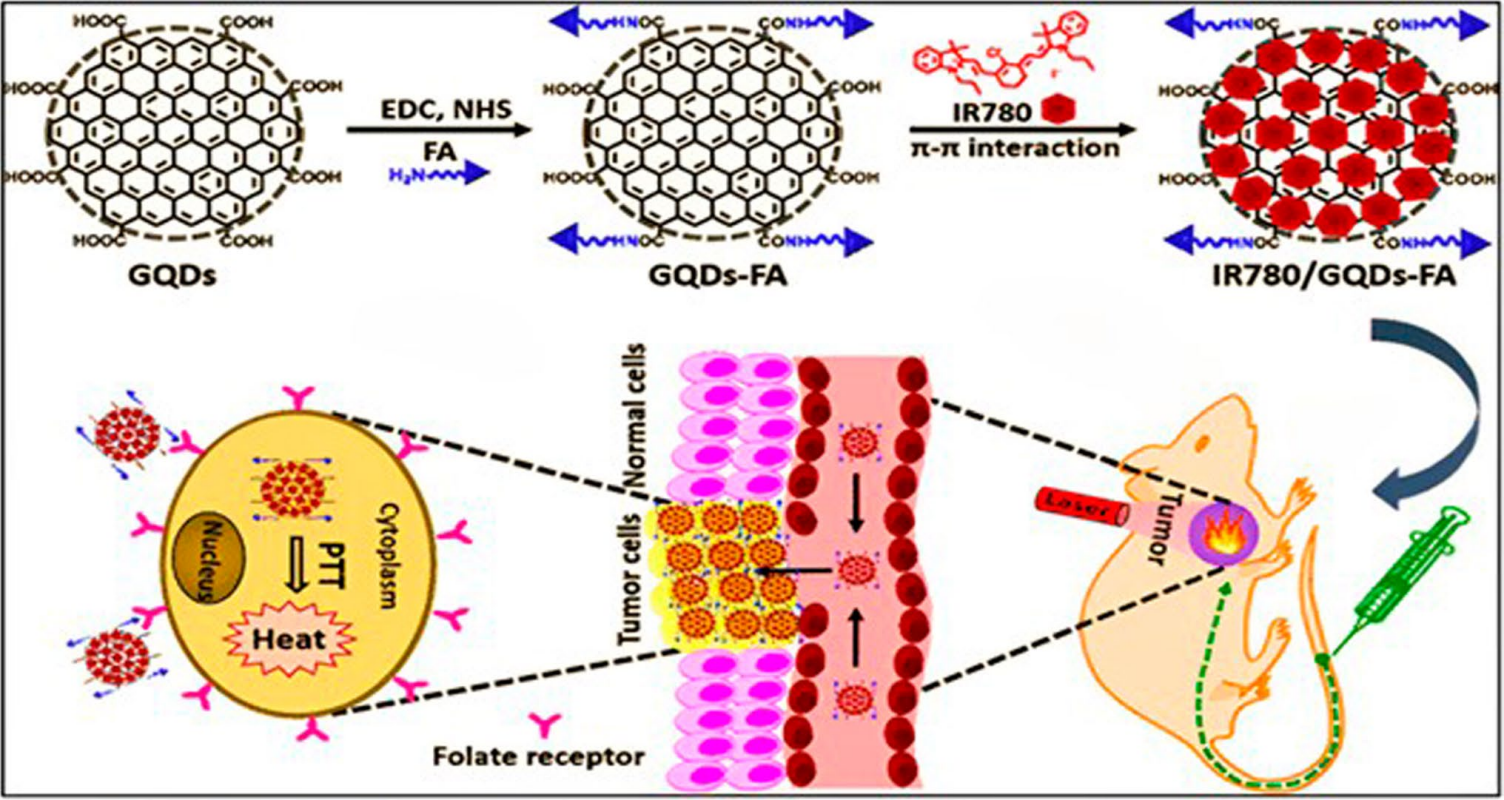
Schematic illustration of targeted PTT with IR780 Iodide on Folic Acid-functionalized GQDs. Reprinted (adapted) with permission from ref [145]. Copyright (2017) American Chemical Society

PTT uses, electromagnetic wave energy with wavelengths in between (700–1350 nm) between the first and second and NIR infrared region and absorbent nanomaterials at these wavelengths, which have a conversion efficiency of light to heat. Thus, by turning light energy into heat and raising the temperature to between 42 and 46 °C, cancer cells are killed through the rupturing of cell membranes and proteins [141]. In addition to its localized tumor ablation capabilities and compatibility with other treatments, PTT boasts minimal invasiveness, reduced side effects, and the potential for targeted delivery, making it a promising avenue for cancer therapy. Furthermore, PTT exhibits tunable parameters, allowing for customization based on specific tumor characteristics and patient needs. GQDs exhibit promising attributes as photothermal materials due to their chemical stability, broad light absorption range, lightweight nature, and cost-effectiveness. Their capacity to convert light into heat relies on the excitation of loosely bound π electrons and subsequent relaxation to these states [142]. Additionally, GQDs are regarded as emerging nanomaterials for PTT in cancer treatment owing to their favourable biological compatibility and quick elimination from organs [143]. Currently, great efforts have been given for GQDs-based nanocomposite for PTT. For instance, Chen L. and his team created a synergetic photo-chemotherapy for hepatocellular carcinoma using aptamer-modified GQDs and magnetic chitosan. PTT temperature climbed swiftly to about 43 °C in the photothermal group, then progressively increased to 48 °C near the tumour site, inhibiting tumour growth and extending the survival time of tumour-bearing mice [107]. Likewise, Tian Z and colleagues reported the development of ZIF-8/GQDs nanocomposite for chemo- and photothermal treatment with drug encapsulation. Additionally, the pH-controlled DOX release and the photothermal effect of the GQDs nanoparticles resulted in a synergistic effect that killed cancer cells [144]. Figure 11 depicts a schematic of the targeted PTT using GQDs-based nanomaterial for the eradication of tumours in both in vitro and in vivo conditions. However, despite promising preclinical results, there are still challenges to address before clinical translation, including optimizing targeting strategies, enhancing tumor accumulation, and ensuring controlled drug release. Additionally, further investigation is needed to understand the long-term biocompatibility and potential toxicity of GQDs in vivo. While recent studies have shown encouraging outcomes with GQDs-based nanocomposites, more rigorous preclinical and clinical studies are necessary to validate their efficacy and safety for widespread clinical use. Overall, while GQDs hold great promise in PTT, continued research efforts and critical analysis are essential to overcome existing challenges and realize their full potential in cancer therapy.

### Photodynamic therapy

PDT is a procedure that involves the use of photosensitizers, which, when activated by light of a certain wavelength, react with molecular oxygen to produce reactive oxygen species (ROS) such as singlet oxygen (^1^O_2_), to target and kill cancer cells in a specific area. These ROS are made by a photosensitizer (PS) when oxygen is present, and light is applied between UV–visible region. Researchers have shown that GQDs can be used for both imaging and treating cancer. As an extra benefit, GQDs make these photosensitizers more photoactive, which means they could be used as helpers in PDT [146, 147]. According to a recent study, Reagen S. and colleagues addressed the use of GQDs-hollow mesoporous silica nanocomposite (hMSNs) for fluorescence imaging for dual cancer treatment combining drug delivery and PDT. The GQDs' ability to maintain their fluorescence is due to the surface attachment to hMSNs. The nanocomposite was used to successfully treat PDT, and they demonstrated induced toxicity with increasing concentration as more oxygen was produced [148]. In another study GQDs-based nanocomposites were utilised in treating cancer via PDT, which caused mitochondria-associated apoptotic cell death in TNBC (MDA-MB-231) cells [149]. Similar to the previous group, this group used microwave-assisted synthesis to create titanium-based GQDs nanocomposite. After being photoactivated with NIR light, the nanocomposites produced ROS, primarily singlet oxygen (^1^O_2_), which significantly increased MDA-MB-231 cell death [149]. Recent research highlights the significance of selecting appropriate photosensitizers for PDT applications. Studies have demonstrated the promising potential of boron-dipyrromethene (BODIPY) with singlet-oxygen production quantum yields of up to 83% when doped with GQDs. However, BODIPY exhibits poor water solubility, limiting its practical utility. Recent investigations have addressed this issue by improving the water solubility of BODIPY through fluorescence resonance energy transfer (FRET). In these studies, BODIPY was incorporated as an acceptor chromophore, with nanoparticles serving as donors, resulting in enhanced PDT efficiency [150–152]. Nevertheless, issues such as selecting the appropriate photosensitizer and ensuring long-term biocompatibility must be solved. Optimising GQD-based nanocomposites for targeted drug administration and monitored release is critical for achieving better therapeutic effects. More research is required to overcome these obstacles and enable the clinical translation of GQDs in PDT.

### Combinational therapies

**Fig. 12:**
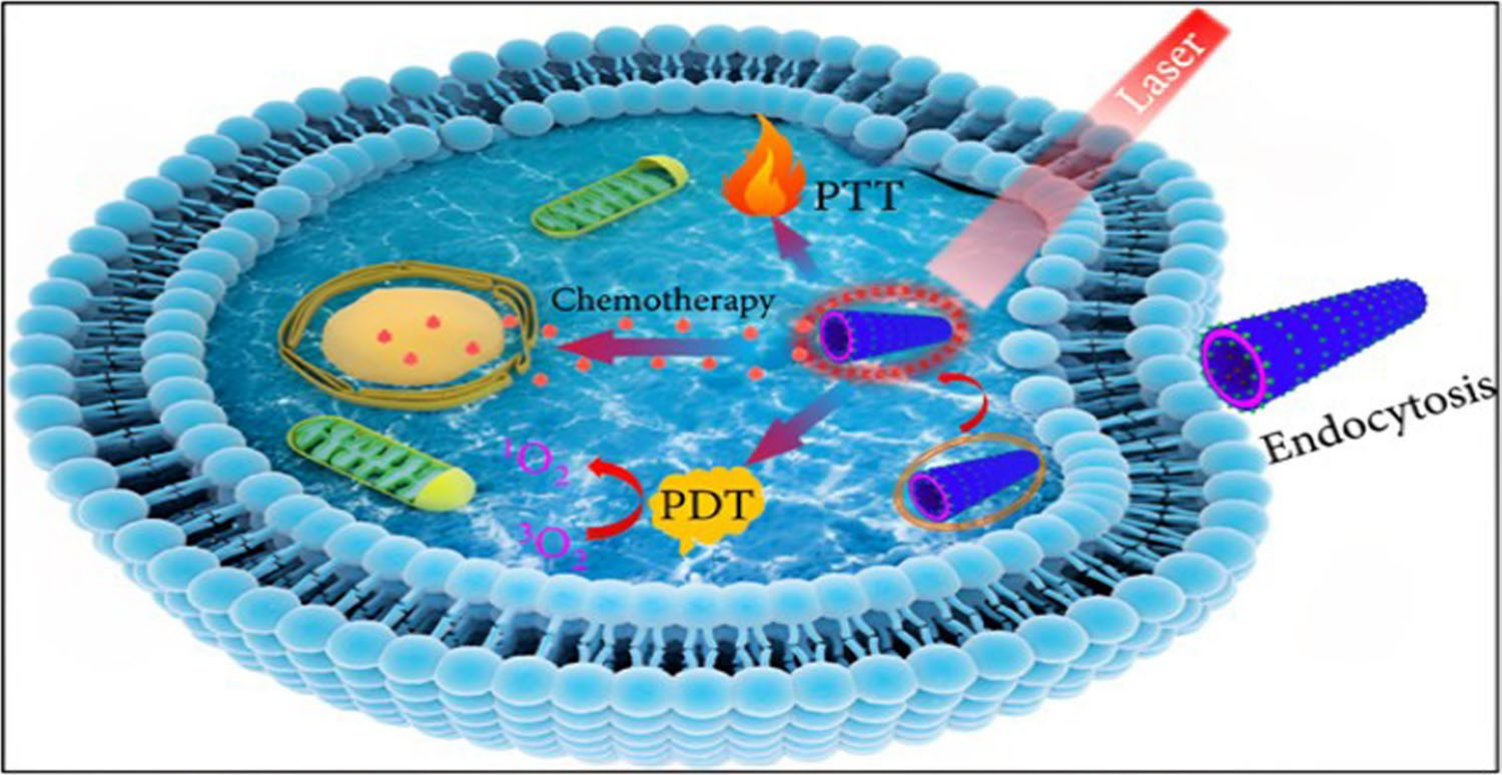
Schematic for chemo-PDT/PTT synergistic therapy of glioma cancer cells. Reprinted (adapted) with permission from ref. [155] Copyright (2021) Xiaoli Cai et al.

Cancer therapy's cornerstone is multiple combination therapy, a method that combines two or more therapeutic therapies. This strategy may decrease the occurrence of drug resistance while also having therapeutic anti-cancer effects, such as lowering tumour growth and cancerous potential, stopping mitotically active cells and causing apoptosis [153]. Yu C. and his team's workers added GE11, an EGFR antagonist peptide, to GQDs and employed it as a drug delivery system for clinical chemotherapy medicines. In vivo, investigations have demonstrated that the specific targeting effect of a nanocomposite enrichment at the tumour location leads to a significant reduction in the proliferation of tumour cells. This novel targeted therapy fluorescence probe offers novel approaches for the investigation of drug release mechanisms and the management of nasopharyngeal cancer [154]. Figure 12 depicts representation of synergistic therapeutic approaches aimed at combating cancer.

Yang et al. employed GQDs to increase PDT and PTT as multifunctional nanocarriers. Additionally, the nanocomposite has increased T1-weighted MRI and deep-red fluorescence that responds to tumours, allowing for imaging of the tumour during therapy. Thereby this potent hybrid nanosystem offered a new multimodal tool for the diagnosis and treatment of cancer [156]. Similarly, using the silica shells, Wo F and colleagues created magnetic nanospheres/GQDs. Mechanical stimulation, photothermal damage, photodynamic toxicity, and chemotherapy were four different treatment strategies used in conjunction with the multimodal system to kill cancer cells [51]. Combining GQDs with various cancer therapies like chemotherapy, PDT, and PTT enhances treatment efficacy by targeting tumours more effectively and reducing drug resistance. However, critical analysis is needed to optimize dosing, minimize side effects, and understand the mechanisms of interaction between GQDs and other therapies for improved cancer treatment outcomes.

## Critical analysis and challenges in clinical translation of GQDs

In addition to the insights presented, it is critical to explore other details from existing research to gain a complete grasp of the issues connected with GQDs. Photoluminescence quenching in GQDs is a complex phenomenon impacted by parameters, including surface defects, functional groups, and chemical contaminants. Surface passivation and defect engineering have been proven in studies to have a considerable impact on photoluminescence characteristics, allowing for the mitigation of quenching effects. Furthermore, while GQDs are generally low in toxicity, there are differences between research due to variations in experimental conditions and GQDs properties. GQDs' biocompatibility is regulated by size, surface chemistry, and exposure concentration. Understanding the root mechanisms of GQD toxicity, such as oxidative stress and cellular absorption pathways, is critical for precisely predicting their behavior in biological systems. Furthermore, long-term in vivo investigations are required to fully assess potential chronic effects and biodistribution profiles. The stability in physiological conditions is critical for the clinical translation of GQDs. Surface modifications with biocompatible polymers improve stability by reducing nonspecific binding and negative interactions with biomolecules. Polymeric coatings such as PEG and polydopamine (pDA) protect GQDs from external effects, whereas natural matrices increase biocompatibility. Long-term stability studies under physiological settings are critical for monitoring GQDs behavior over time, assuring safe and effective therapeutic use [111, 113, 157, 158].

Several significant hurdles hinder the clinical translation of GQDs in a variety of biomedical applications. To begin, the high variety among GQDs synthesized using various methods leads in variable physicochemical properties, making standardization challenging. Second, the potential toxicity of GQDs, such as generating inflammatory responses and DNA damage, raises questions about their safety in clinical settings. Furthermore, GQDs have limited PDT efficiency in their pure form, requiring precision engineering to improve their efficacy. Furthermore, the absence of thorough studies on genotoxicity and its long-term effects on normal tissues emphasizes the importance of meticulous monitoring and toxicity mitigation techniques. Furthermore, there is a need for novel ways to increase GQD selectivity, clearance rate, and therapeutic efficacy for better clinical results. Overall, while GQDs show enormous promise for biomedical applications, overcoming these issues is critical to their successful clinical translations [159–161].

## Summary and outlook

The integration of GQDs with diverse materials has proven to be a promising strategy for the development of highly effective biomedical applications addressing various health challenges. Despite the substantial progress and rapid expansion of GQDs-based nanocomposites, they are still largely in their early stages of development. In this review, we have underscored emerging and alternative methods for the efficient fabrication of GQDs-based nanocomposites, highlighting their interactions with different materials within the biomedical realm. Our exploration has delved into recent remarkable advancements in the biomedical field facilitated by GQDs-based nanocomposites. The diverse characteristics exhibited by these nanocomposites, stemming from varied preparation methods, grant them access to a wide spectrum of physicochemical properties. However, mitigating toxicity and enhancing the clearance rate of GQDs within organs, crucial for minimizing harm to healthy tissues, necessitate strategies such as size management and improved selectivity. These strategies may involve variations in preparation methods, choice of elements, and other influencing factors. Undoubtedly, a paramount focus should be placed on comprehending and revealing the interactions of GQDs-based nanocomposites with biological systems through extensive biological assessments and standardized protocols. The future applications of GQDs-based nanocomposites demand further research to unravel the intricate interactions occurring between the nanomaterials and GQDs, as these interactions profoundly impact the properties of the resulting nanocomposites. While extensive research on these interactions holds the potential for versatile applications, including targeted drug delivery, PTT, PDT, biosensing, and bioimaging, the precise design of their properties remains a challenging and significant undertaking. In conclusion, we foresee a promising future for the continued development of GQDs-based nanocomposites, offering solutions to a variety of unmet therapeutic challenges in the biomedical field.

## Data Availability

Not applicable.
